# Radio-sensitization effect of an mTOR inhibitor, temsirolimus, on lung adenocarcinoma A549 cells under normoxic and hypoxic conditions

**DOI:** 10.1093/jrr/rrv021

**Published:** 2015-04-16

**Authors:** Hiroki Ushijima, Yoshiyuki Suzuki, Takahiro Oike, Mayumi Komachi, Yuya Yoshimoto, Ken Ando, Noriyuki Okonogi, Hiro Sato, Shin-ei Noda, Jun-ichi Saito, Takashi Nakano

**Affiliations:** Department of Radiation Oncology, Gunma University Graduate School of Medicine, 3–39–22 Showa-machi, Maebashi, Gunma, 371–8511, Japan

**Keywords:** hypoxia, radiation, mTOR, HIF-1α, radio-sensitizer

## Abstract

The mammalian target of rapamycin (mTOR) correlates with cell survival under hypoxia and regulates hypoxia-inducible factor-1α (HIF-1α), a key protein in hypoxia-related events. However, the role of mTOR in radio-resistance has not been fully investigated. Therefore, the effect of mTOR on the radio-resistance of cancer cells under hypoxia was evaluated using the mTOR inhibitor temsirolimus. Clonogenic survival was examined in the A549 human lung adenocarcinoma cell line under normoxia or hypoxia, with or without temsirolimus. An oxygen enhancement ratio (OER) was calculated using the D_10_ values, the doses giving 10% survival. Western blotting was performed to investigate the effect of temsirolimus on mTOR and the HIF-1α pathway under normoxia and hypoxia. A549 cells showed a radio-resistance of 5.1 and 14.2 Gy, as indicated by D_10_ values under normoxia and hypoxia, respectively; the OER was 2.8. The cell survival rates under hypoxia and with temsirolimus remarkably decreased compared with those under normoxia. The D_10_ values of the cells under normoxia and hypoxia were 4.8 and 5.4 Gy, respectively (OER = 1.1). mTOR expression was suppressed by temsirolimus under both normoxia and hypoxia. HIF-1α expression decreased under hypoxia in the presence of temsirolimus. These results suggest that temsirolimus can overcome the radio-resistance induced by hypoxia. When the fact that mTOR acts upstream of HIF-1α is considered, our data suggest that the restoration of radiation sensitivity by temsirolimus under hypoxia may be associated with the suppression of the HIF-1α pathway. Temsirolimus could therefore be used as a hypoxic cell radio-sensitizer.

## INTRODUCTION

There is growing evidence that hypoxia in the microenvironment of a solid tumor negatively impacts radiotherapy [1–4]. A number of reports have confirmed the existence of hypoxic cells in human cancers using an Eppendorf probe, which can directly measure intratumoral oxygen pressure (pO_2_), and have shown that hypoxia in tumors correlates with a worse clinical outcome [5–7]. We have also reported that low intratumoral pO_2_ before and during radiation therapy correlates with a poor local control rate in cervical cancer patients treated with radiotherapy alone [8].

The mammalian target of rapamycin (mTOR) is a downstream protein of the phosphatidylinositol 3-kinase (PI3 K)/Akt pathway [9]. mTOR is strongly associated with proliferation and cell survival, and mTOR inhibitors have been used in clinical applications as a molecular target drug against malignant tumors, such as renal cell carcinomas [10–12]. Several studies have shown that mTOR inhibitors have radio-sensitizing effects on a number of malignant tumors, such as breast cancer [13, 14]. Recently, research on mTOR has focused on its role in promoting cell survival under hypoxia, and mTOR is thought to regulate the expression of hypoxia-inducible factor-1α (HIF-1α) under hypoxia [15]. HIF-1α is induced under hypoxia and plays an important role in tumor progression by upregulating genes that control angiogenesis, metastasis, and resistance to oxidative stress [16]. HIF-1α overexpression in tumor cells correlates with worse clinical outcomes following radiation therapy [17, 18]. Based on these reports, we hypothesized that mTOR plays an important role in radio-resistance under hypoxia. We evaluated the correlation between the inhibition of mTOR and HIF-1α expression, focusing on the radio-sensitizing effects of the mTOR inhibitor temsirolimus under normoxia and hypoxia.

## MATERIALS AND METHODS

### Cell line and cultures

The human lung adenocarcinoma cell line A549 was purchased from RIKEN BRC (Tsukuba, Japan) through the National Bio-Resource Project of the Ministry of Education, Culture, Sports, Science and Technology, Japan. A549 cells were cultured in RPMI-1640 (Invitrogen, Carlsbad, CA, USA) supplemented with 10% fetal bovine serum (BioWest, Miami, FL, USA) and 5% penicillin–streptomycin (Gibco, Grand Island, NY, USA) in a humidified incubator at 37°C and 5% CO_2_.

### Reagents

The mTOR inhibitor temsirolimus was purchased from Pfizer (New York, NY, USA) and was dissolved in its accompanying diluent to a stock concentration of 10 mM and stored at –20°C. This stock solution was diluted to reach a final concentration of 0.01–10 nM prior to use with culture media, and the cells were exposed to the drug for 48 h prior to clonogenic assays and western blot analyses.

### Irradiation

The cells were irradiated with 200 kV X-ray via RX-650 (Faxitron Bioptics, Tucson, AZ, USA) at a dose rate of 0.7 Gy/min without a filter under both normoxic and hypoxic conditions in the hypoxic chamber described below.

### Induction of hypoxia

To expose cells to hypoxia, a hypoxic chamber with an atmospheric gas concentration control apparatus designed by Furusawa *et al.* [19] was used. The cells were incubated in a hypoxic chamber that was flushed with 5% CO_2_ and 95% nitrogen, and the pO_2_ of culture media reached <0.1 mmHg (<0.00013% O_2_) about 30–60 min from hypoxia induction. The oxygen concentration was assessed in real time using an oxygen electrode (UOE-04 T, Unique Medical, Tokyo, Japan). The details are reported elsewhere [20].

### Clonogenic survival assays

**Fig. 1.**
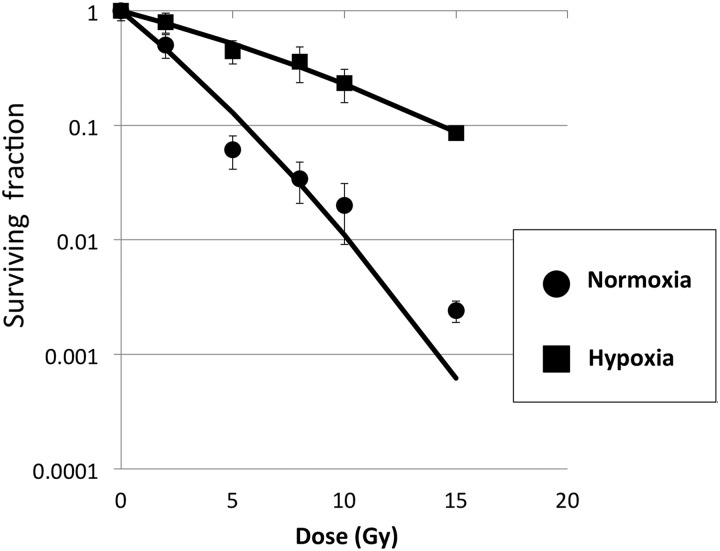
Clonogenic survival curves of A549 cells under normoxia and hypoxia of pO_2_ < 0.1 mmHg for 24 h. Each plot represents the average of the survival fraction with the standard deviation. The plots were fitted using the linear–quadratic model from all of the data. The D_10_ values were 5.1 and 14.2 Gy, respectively. The OER was calculated as 2.8. D_10_ = dose at which 10% of the cells survive, OER = oxygen enhancement ratio, pO_2_, oxygen partial pressure.

Clonogenic survival assays were performed to calculate the cell survival fraction of the following groups: (i) X-ray irradiation alone (under normoxia and hypoxia); (ii) a single temsirolimus treatment; and (iii) X-ray irradiation combined with temsirolimus (under normoxia and hypoxia). The procedure schematics are shown in Fig. 1a–c. The cultured A549 cells were trypsinized, counted, and seeded at a concentration of 1 × 10^5^ cells onto glass dishes and were then incubated at 37°C for 24 h. For the temsirolimus treatment, the cells were exposed to temsirolimus (0.01, 0.1, 1 and 10 nM for temsirolimus alone, 1 nM for temsirolimus with X-ray irradiation) for another 48 h. For hypoxic conditions, the cells were incubated in the hypoxic chamber described above for 24 h before X-ray irradiation and an additional 1 h after irradiation. With the exception of temsirolimus single treatments, the cells were irradiated with 2, 5, 8, 10 and 15 Gy of X-rays 72 h after cell seeding. The temsirolimus-treated and/or irradiated cells were then trypsinized, counted, reseeded in triplicate to 6-well plates at a specified number with fresh medium, and incubated at 37°C for 10 days under normoxia to form colonies. The cells were fixed and stained with 2% crystal violet (Sigma–Aldrich, St Louis, MO, USA) solution in 100% ethanol. Colonies containing at least 50 cells were counted. The fraction of the cells that survived treatment was calculated as the ratio of that to the non-treated control (plating efficiency). Averages and SDs were calculated from three separate experiments.

### Evaluation of the oxygen enhancement ratios

An oxygen enhancement ratio (OER) was calculated for the dose at which 10% of the cells survived (D_10_). The D_10_ value was calculated from each survival dataset via a curve-fitting method using the following linear–quadratic (LQ) model:

$$\text{SF }=\text{exp}(-\alpha \text{D }-\beta {\text{D}}^{2}),$$

where SF is the surviving fraction and D is the irradiation dose. In this LQ model, an alpha/beta value = 10 was used, as previously reported by our group [20].

### Western blot analysis

A549 cells were washed with phosphate-buffered saline and scraped from culture plates on ice. Whole cell lysates and nuclear protein were extracted using a cell lysis buffer (Millipore, Billerica, MA, USA) supplemented with a proteinase inhibitor cocktail and phosphatase inhibitor cocktail (Roche Diagnostics, Indianapolis, IN, USA). The protein concentrations of the collected supernatants were quantified using the BCA Protein Assay Kit (Pierce, Rockford, IL, USA). Equal amounts of protein were electrophoresed in 4–15% Mini-Protean TGX gels (Bio-Rad Laboratories Inc., Hercules, CA, USA) and then transferred onto PVDF membranes (Bio-Rad Laboratories Inc.) using the Trans-Blot Semi-Dry system. (Bio-Rad Laboratories Inc.). The blots were blocked in PBS (−) with 0.05% Tween 20 (Amersham Biosciences Corp., Piscataway, NJ, USA) buffer (PBST) with 1% BSA. The membranes were incubated with primary antibodies at 4°C overnight, followed by incubation with secondary antibodies at room temperature for 1 h using Can Get Signal® Immunoreaction Enhancer Solution (Toyobo, Tokyo, Japan). The primary antibodies for mTOR, phosphorylated mTOR (p-mTOR and Ser2448), phosphorylated p70 S6 kinase (p-p70S6k and Thr389), and phosphorylated 4E-BP1 (p-4E-BP1 and Thr37/46) were purchased from Cell Signaling Technology (Danvers, MA, USA), and HIF-1α and β-actin were purchased from Novus Biological (Littleton, CO, USA) and Sigma–Aldrich, respectively. The secondary antibodies, horseradish peroxidase-conjugated rabbit anti-mouse IgG and goat anti-rabbit IgG, were obtained from DakoCytomation (Glostrup, Denmark). The western blots were visualized using ECL plus reagents and an ImageQuant LAS 4000 system (GE Healthcare, Piscataway, NJ, USA). Quantification was performed using ImageJ version 1.48 (http://rsbweb.nih.gov/ij/, National Institutes of Health, Bethesda, MD, USA).

## RESULTS

### A549 cells show radio-resistance under hypoxic conditions

Radio-resistance against X-ray irradiation was evaluated under hypoxia. As shown in Fig. 1, A549 cells under hypoxia showed radio-resistance compared with those under normoxia. The D_10_ values of the cells under normoxia and hypoxia were 5.1 and 14.2 Gy, respectively, resulting in an OER of 2.8.

### Temsirolimus decreases the survival fraction of A549 cells

**Fig. 2.**
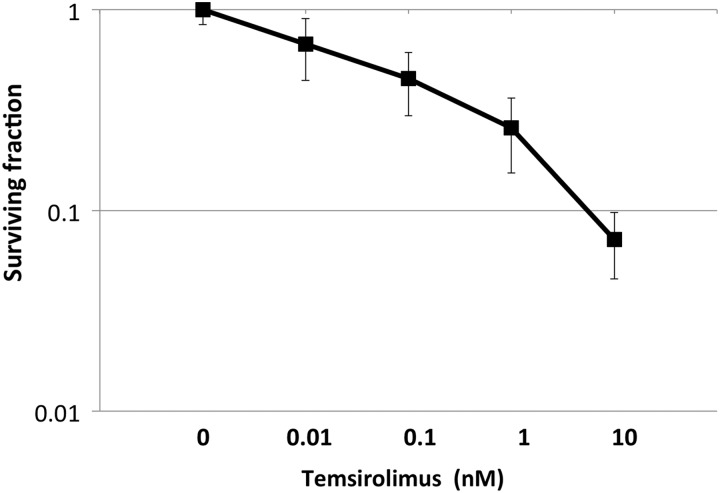
Clonogenic survival curves of A549 cells treated with the indicated concentrations of temsirolimus for 48 h under normoxia. The IC_50_ value was 0.08 nM.

The cell survival rates of the cells exposed to temsirolimus single treatments were evaluated using clonogenic survival assays to identify the optimal concentration of temsirolimus to use in combination with X-ray irradiation. Figure 2 showed that temsirolimus decreased the survival fraction of A549 cells in a dose-dependent manner. The IC_50_ of temsirolimus was 0.08 nM, and the survival fraction at 1 nM was 25%. Based on these data, a final concentration of 1 nM temsirolimus was used with X-ray irradiation in subsequent experiments. This concentration of temsirolimus is much lower than maximum drug concentration (Cmax) in clinical use [21].

### Temsirolimus decreases radio-resistance under hypoxic conditions

**Fig. 3.**
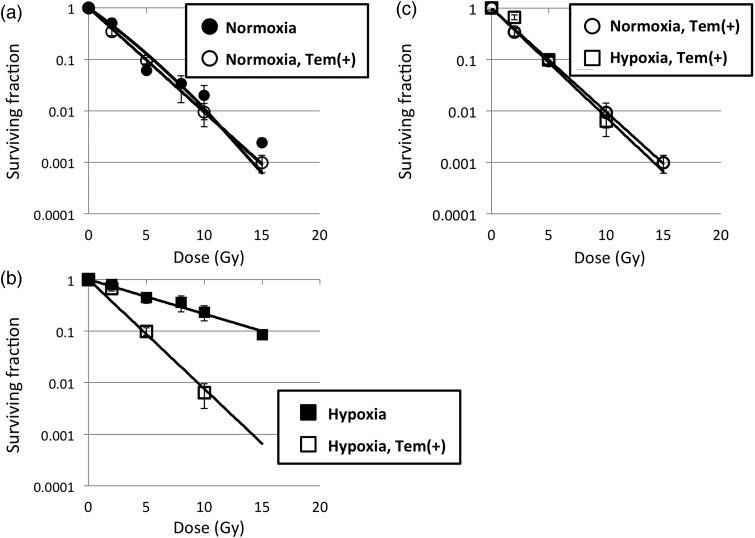
Clonogenic survival curves of A549 cells irradiated with temsirolimus. **(a**) X-ray irradiation with or without temsirolimus under normoxia. Survival curves are shown as the ratio to the control [0 Gy, temsirolimus (−), under normoxia]. (**b**) X-ray irradiation with or without temsirolimus under hypoxia for 24 h. Survival curves are shown as the ratio to the control [0 Gy, temsirolimus (−), under hypoxia]. (**c**) X-ray irradiation with temsirolimus under normoxia and hypoxia. The D_10_ values of the cells under normoxia and hypoxia were 4.8 and 5.4 Gy, respectively. The OER was calculated as 1.1. D_10_ = dose at which 10% of the cells survive, OER = oxygen enhancement ratio.

**Fig. 4.**
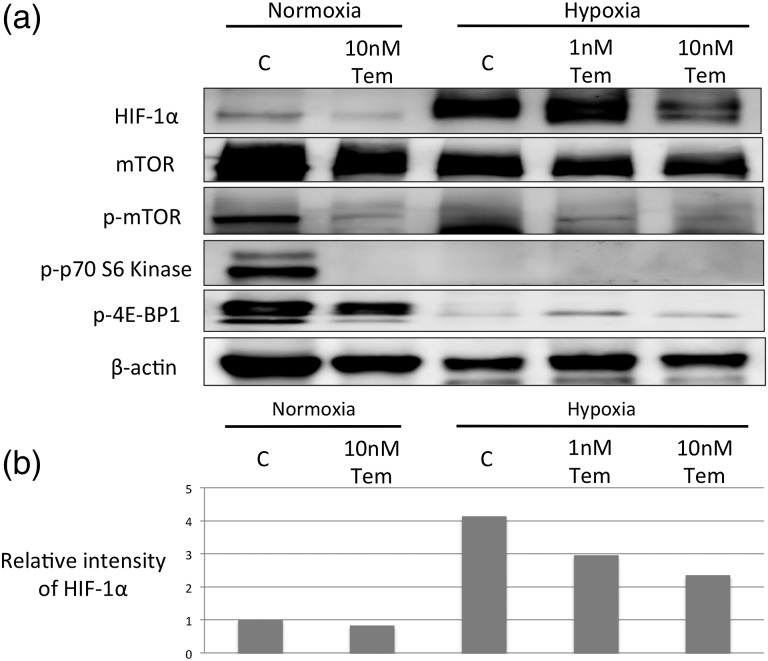
Effect of temsirolimus on A549 cells under normoxia and hypoxia. (**a**) Levels of HIF-1α, p-mTOR, mTOR, p-p70S6k and p-4E-BP1 were examined using western blotting. β-actin is shown as a loading control. (**b**) Bar graph showing the relative band intensities for HIF-1α to β-actin (control). The data are shown after normalization to samples on normoxia without temsirolimus. The experiment has been done three times with identical results. Representative images of western blot are shown. HIF-1α = hypoxia-inducible factor-1α, mTOR = mammalian target of rapamycin, p-mTOR = phosphorylated mTOR, p-p70S6k = phosphorylated p70 S6 kinase, p-4E-BP1 = phosphorylated 4E-BP1.

Changes in the cell survival rate with temsirolimus were evaluated under normoxia and hypoxia in combination with X-ray irradiation. Under normoxia, the radio-sensitizing effect of temsirolimus could not be detected (Fig. 3a). However, under hypoxia, the cell survival rates of the A549 cells were remarkably and synergistically decreased when irradiation was combined with temsirolimus (Fig. 3b). Figure 4c shows the survival fractions of A549 cells treated with a combination of X-ray and temsirolimus treatments under normoxia and hypoxia. The D_10_ values of the cells under normoxia and hypoxia were 4.8 and 5.4 Gy, respectively, and the OER was 1.1.

### Temsirolimus inhibits the mTOR pathway under normoxia and decreases HIF-1α expression under hypoxia

The effects of temsirolimus on the mTOR pathway and HIF-1α in A549 cells were analyzed using western blot analysis (Fig. 4). Under both normoxia and hypoxia, the expression levels of both mTOR and p-mTOR were similarly suppressed by temsirolimus treatment in a dose-dependent manner, and p-mTOR expression was more significantly suppressed than that of mTOR.

HIF-1α expression was not observed under normoxia. However, the cells exposed to hypoxia for 24 h expressed higher levels of HIF-1α, and HIF-1α expression decreased with temsirolimus treatment in a dose-dependent manner. The expression of p-p70S6k and p-4E-BP1 were examined as surrogate markers for mTOR activation. p-p70S6k and p-4E-BP1 expression were not observed under hypoxia and were suppressed via temsirolimus treatment under normoxia.

## DISCUSSION

mTOR is a downstream target of the PI3 K/Akt pathway and has been shown to affect numerous cells and processes such as proliferation, apoptosis and autophagy [9]. Therefore, mTOR has been a molecular target of cancer therapy, and its inhibitors (such as rapamycin and its analogs) are used as molecular target drugs. Furthermore, Albert *et al.* and Chang *et al.* reported that mTOR inhibitors imparted radio-sensitizing effects [13, 14]. However, because they did not analyze radio-sensitization in terms of hypoxia, its detailed mechanisms remain unclear. Shinohara *et al.* showed that irradiation activated the expression of EGFR downstream of mTOR signaling, including its expression in the vascular endothelium. Therefore, the authors concluded that the radio-sensitizing effects of mTOR inhibitors may have derived from the suppression of tumor angiogenesis [22].

In the present study, there were no significant differences in the cell survival rates after irradiation alone and irradiation with temsirolimus under normoxia. In contrast, under hypoxia, the cell survival rate after irradiation significantly decreased in the presence of temsirolimus, and the cytotoxic effect appeared to be synergistic. In addition to the suppression of mTOR and expression of p-mTOR by temsirolimus under both normoxia and hypoxia, a decrease in HIF-1α expression was also observed under hypoxia after 24 h, which was intended to simulate chronic hypoxia.

Under hypoxia, HIF-1α is a key protein that affects cell survival by promoting ATP metabolism and proliferation [15, 16]. Additionally, HIF-1α overexpression correlates with resistance against radiotherapy in clinical situations [17, 18]. Therefore, HIF-1α has been suggested as the molecular target of radio-sensitivity. In a review article, Bradly *et al.* stated that mTOR acts upstream of HIF-1α and is involved in the HIF-1α pathway [15], and Sarbassov *et al.* reported that HIF-1α expression was suppressed by the inhibition of the mTOR pathway. Moreover, the mTOR inhibitor rapamycin suppressed HIF-1α expression under hypoxia for 24 h, which is in accordance with our results [23]. These data suggest that the restoration of radiation sensitivity in the present study may have resulted from the modulation of the HIF-1α pathway via the inhibition of mTOR.

p-p70S6k expression was suppressed under hypoxia, which was in agreement with previous reports [24, 25]. This result may suggest that under normoxia, the radio-sensitivity of cells is strongly controlled by the mTOR pathway but is unrelated to the HIF-1α pathway because HIF-1α expression is typically very low under normoxia. However, under hypoxia, the radio-sensitivity of cells may be more strongly affected by the HIF-1α pathway than the mTOR pathway because the expression of p-p70S6k and p-4E-BP1 were originally very low, and HIF-1α expression was high under hypoxia.

We previously reported a correlation between HIF-1α and radio-resistance under acute hypoxia in A549 cells [20]. In that report, cells were exposed to hypoxia for 1 h to investigate the correlation between radio-resistance and HIF-1α expression (we simulated acute hypoxia). HIF-1α expression was suppressed by a HIF1-α inhibitor, YC-1, although no significant difference was observed between the survival fraction of cells irradiated with or without YC-1 in clonogenic survival assays. Therefore, we hypothesized that HIF-1α may not be the immediate cause of acute hypoxia-induced radio-resistance in A549 cells. The time that cells spend under hypoxia may be relevant to radio-resistance observed during hypoxia.

mTOR is a key component of two protein complexes, mTORC1 and mTORC2 [23]. Potiron *et al.* reported that the dual PI3 K/mTOR inhibitor BEZ235 imparts a radio-sensitizing effect on prostate cancer cells under normoxia and hypoxia; the effect observed under hypoxia was also synergistic [26], which was in agreement with our results. Despite synergistic radio-sensitization under hypoxia, HIF-1α suppression was not observed in this report. BEZ235 inhibits both mTORC1 and mTORC2 in addition to PI3 K. However, temsirolimus inhibits only mTORC1 [27]. mTORC1 affects HIF-1α more directly than mTORC2 [28]. Our data and these reports suggest that mTORC1 suppression may lead to significant radio-sensitizing effects under hypoxia. It has also been suggested that another molecule in the PI3 K pathway (other than HIF-1α) imparts a radio-sensitizing effect under hypoxia.

Furthermore, autophagy and synthetic lethality in relation to mTOR and hypoxia [29, 30] may also play roles in radio-sensitization. Therefore, the mechanisms behind the synergistic effect of temsirolimus under hypoxia remain unclear. Further experiments will be necessary to clarify the mechanisms of radio-sensitization. In radiation therapy, a therapeutic gain between tumors and the surrounding normal tissues is important for achieving local control with fewer radiation-induced complications. Regarding the oxygen tension of various tissues, most normal tissues are in normoxia, whereas bulky tumors consist of remarkably hypoxic cell populations. Therefore, the synergistic enhancement of radiation sensitivity under hypoxia with the administration of temsirolimus leads to the selective radio-sensitization of tumors and increases the therapeutic gain between tumors and surrounding normal tissues, which results in better control of radiation therapy.

The present study examined the radio-sensitivity of A549 cells under normoxia and hypoxia using the mTOR inhibitor temsirolimus. The mTOR pathway and HIF-1α expression under hypoxia were suppressed in A549 cells by temsirolimus, and the OER decreased from 2.8 to 1.1 in the presence of temsirolimus. Furthermore, the radio-sensitization effects of temsirolimus under hypoxia were synergistic in restoring a level of radio-sensitivity similar to that observed under normoxia. Therefore, temsirolimus is a promising hypoxic radio-sensitizer and a molecular target anti-cancer drug that warrants further investigation.

## FUNDING

Funding to pay the Open Acces publication charges for this article was provided by Hiroki Ushijima.
